# Supplementary material for: Placental transfer of Letermovir & Maribavir in the ex vivo human cotyledon perfusion model. New perspectives for in utero treatment of congenital cytomegalovirus infection
Source: PLoS One. 2020 Apr 30;15(4):e0232140. doi: 10.1371/journal.pone.0232140 (PMC7192425; doi:10.1371/journal.pone.0232140)
Supplement: Attachment — Submitted filename: Response 2 to reviewers.docx [file pone.0232140.s003.docx]

Reviewer #1: Thank you for addressing the previous comments. The manuscript is improved and nearly suitable for publication. The only changes I request regard Figure 1. In my previous review I had asked that Figure 1 (Letermovir) and Figure 2 (Maribarvir) be merged into a single figure denoting panel A and B respectively, which the authors have done. However, the graphs were also merged (not requested), which creates confusion as a single graph represents only one panel but is labeled A and B. Please replace with the original two graphs and label Letermovir as A and the Marbarvir as B in vertical order.

It was done.

Also annotate the results section according, line 139 should read "Letermovir (Table 3, Fig 1A)" and line "Maribavir (Table 3, Fig 1B".

It was done.

Finally, please remove the abbreviations from the titles as indicated in my previous sentence.

It was done.

Once these minor changes are made, it is this reviewer’s opinion that the manuscript is ready for publication.

We thank the reviewer for this comment.

Reviewer #2: 1. Regarding author's response concerning the concentration of albumin. I accept their response. Yet a comment about this specific issue should be added, as they commented in here in their own wards: "It is likely that fetal levels of free drugs in vivo will be lower than the values that were obtained in our perfusion experiments, because most of the drug will remain bound to serum albumin in the maternal circulation. It is difficult and impractical to exactly mimic physiological protein binding during the perfusion experiment".

It was done.

2. Regarding author's response about the small study sample. I accept their response.

We thank the reviewer for this comment.

Please add a comment in the study's limitations part, about the relative small study sample of the study.

We added in the limitation part : “Another limitation is the relative small study sample of our study. However, studies published by other experimented teams also evaluated placental transfer of drugs on small studies samples(25,29–31). We only used pure and titrated new drugs and human serum albumin, so the price of each experiment was very expensive. This high-cost was the reason for not doing more experiments.”

3. Regarding the comment about measurements of placental viability and integrity during the periods of perfusion. I accept their response

We thank the reviewer for this comment.
